# Cannabis and cognitive deficiency: a descriptive study

**DOI:** 10.1192/j.eurpsy.2023.1186

**Published:** 2023-07-19

**Authors:** M. Kacem, W. Bouali, M. Henia, S. Brahim, L. Zarrouk

**Affiliations:** Psychiatric department, Taher Sfar Hospital of Mahdia, Mahdia, Tunisia

## Abstract

**Introduction:**

Cannabis is the most widely used illicit drug; 3.8% of the world’s population consumes cannabis on a regular basis. Cannabis use–associated alterations in the domain of cognition have been extensively studied.

**Objectives:**

To research memory deficiency in the young consumers of cannabis in Tunisia.

**Methods:**

This is a transversal descriptive study conducted during two months (January and February 2022). The research involved about 137 participants aged between 18 and 35 years old, exhaustively recruited amid emergency patients of Mahdia Hospital regardless of the reason for their health care seeking. The patients were declared as consumers of cannabis and accepted to be part of this study. Therefore, Data were collected on a pre-determined data sheet that included various information (age, sex, lifestyle, personal and family psychiatric history, age when first used cannabis and the rate of cannabis use …).

Principally, a Functional Impact Assessment (ERF: French abbreviation for *échelle d’évaluation des Répercussions Fonctionnelles* ) scale was used to assess and review working memory.

**Results:**

In our study population, there was a noticeable male predominance of 71%. Hence, the age structure ranged between 18 years old and 35 years old. Among the latters, 65.9% were single, and 29.7% experienced school failure. In this sample, 23.2% had a psychiatric history like depression, bipolarity, etc. The average age of the first use of cannabis was between 18 and 25 years old in 70% of cases.

Besides, a high percentage of association of other substances was found among cannabis users as follows: use of alcohol 72.5%, tobacco 74.6%, ecstasy 41.3%, and cocaine 25.4%. First and foremost, the use of cannabis was considered as a means of indulgence for 66.7% of the study population, as an anxiolytic for 26.8%, and as a sedative for 23.9%.

Additionally, the effect of cannabis use on working memory deficiency according to the functional impact assessment scale was: no deficiency in 19% of cannabis users, minimal in 34%, mild in 32%, moderate in 9%, fairly severe in 4%, very severe in 1%, and extreme in 1% of cases.

More importantly, the percentage of consumers with significant memory deficiency (moderate to extreme) was 15%.

**Conclusions:**

The assumption of the effect of cannabis on memory and cognitive deficiency remains controversial and leads us to suggest further in-depth study of this subject.

**Disclosure of Interest:**

None Declared

